# shinyCircos‐V2.0: Leveraging the creation of Circos plot with enhanced usability and advanced features

**DOI:** 10.1002/imt2.109

**Published:** 2023-05-08

**Authors:** Yazhou Wang, Lihua Jia, Ge Tian, Yihan Dong, Xiao Zhang, Zhengfu Zhou, Xiang Luo, Yang Li, Wen Yao

**Affiliations:** ^1^ National Key Laboratory of Wheat and Maize Crop Science, College of Life Sciences Henan Agricultural University Zhengzhou China; ^2^ Henan Institute of Crop Molecular Breeding Henan Academy of Agricultural Sciences Zhengzhou China; ^3^ College of Agriculture Henan University Kaifeng China

**Keywords:** Circos, data visualization, R/Shiny, shinyCircos, web application

## Abstract

We previously developed shinyCircos, an interactive web application for creating Circos diagrams, which has been widely recognized for its graphical user interface and ease of use. Here, we introduce shinyCircos‐V2.0, an upgraded version of shinyCircos that includes a new user interface with enhanced usability and many new features for creating advanced Circos plots. To help users get started with shinyCircos‐V2.0, we provide detailed tutorials and example input data sets. The application is available online at https://venyao.xyz/shinyCircos/ and https://asiawang.shinyapps.io/shinyCircos/, or can be installed locally using the source code deposited in GitHub (https://github.com/YaoLab-Bioinfo/shinyCircos-V2.0).

## Highlights

- We presented a web application shinyCircos‐V2.0 for creation of a Circos plot.
- shinyCircos‐V2.0 is an upgraded version of shinyCircos with a more user‐friendly interface and many new features.
- Detailed tutorials and example input data sets are provided to enhance the usability of shinyCircos‐V2.0.

## Graphical abstract

We developed a web application called shinyCircos‐V2.0 for creating Circos plots. The process of creating a Circos plot with shinyCircos‐V2.0 involves three simple steps: uploading the input data, setting the plotting parameters, and generating the Circos plot. Our application offers a wide range of plotting parameters that can be used to enhance the appearance of the resulting Circos plot. Overall, shinyCircos‐V2.0 simplifies the creation of Circos plots and provides researchers with a powerful tool for data visualization.

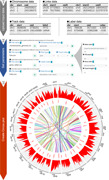

## INTRODUCTION

**Figure 1:**
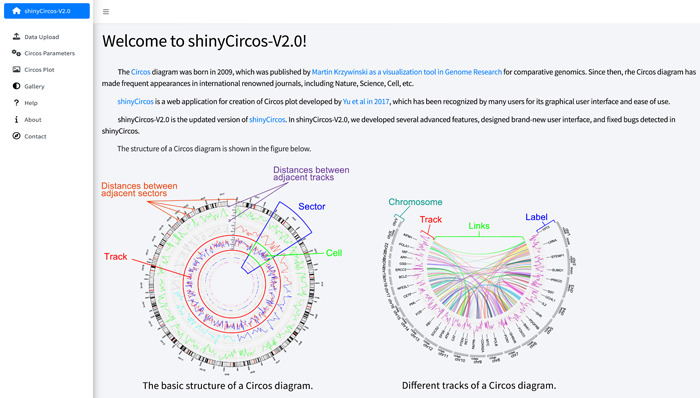
Basic structure of a Circos plot featured on the homepage of the shinyCircos‐V2.0 application. The introductory text at the top of the main panel provides a concise overview of Circos and shinyCircos. The two images at the bottom of the main panel illustrate the basic structure of a Circos plot.

Circos [1] is a visualization tool developed by Krzywinski et al. in 2009 for comparative genomics, which has become an indispensable tool for newly sequenced genomes and other genomic studies. It enables the visualization of various types of genomic data, including single nucleotide polymorphisms, InDels, genes, DNA methylation, and others, in a circular format. Since its development, the Circos plot has been frequently utilized to demonstrate similarities or differences among diverse genomic features associated with the same genomic regions. A typical Circos plot consists of multiple concentric tracks, each representing a different aspect of genomic data (Figure 1). Each track is divided into multiple sectors, which are organized in a circular manner, representing different genomic regions. Within each sector, genomic data can be represented as cells, which are rectangles representing a single region or feature. The outermost track of a Circos plot usually represents the chromosome ideogram or karyotype, displaying the physical positions of chromosomes. Paired genomic regions can be connected by curved lines, designated as links, within the innermost track.

Circos was initially created as a command‐line tool using the Perl programming language [1]. Due to this, installation and configuration of the tool posed a challenge for nonprogrammers. Nonetheless, the Circos tool gained rapid popularity for visualizing genomics data. This popularity led to the development of other tools, such as BioCircos.js [2], Circleator [3], circlize [4], CIRCUS [5], interacCircos [6], NG‐Circos [7], and Galactic Circos [8], for creating Circos plots using various programming languages. Among these, circlize, developed using the R programming language, has received considerable recognition from users due to its clear syntax and powerful functionality. With the aid of the robust graphics systems of R, Circos plots generated using circlize are visually striking.

To enhance the efficiency of Circos plot creation, several tools with graphical user interfaces have been developed, including J‐Circos [9], shinyCircos [10], and the advanced Circos plot module in TBtools [11]. These tools have significantly facilitated the use of Circos plots for data visualization in various fields, including biological studies. Among these tools, shinyCircos (hereafter denoted as shinyCircos‐V1) is an interactive web application developed using R/Shiny as the framework and circlize as the plot engine [4, 10, 12]. As far as we are concerned, shinyCircos is the only web application available online for creating Circos plots, which can also be installed on local computers.

Since its release, shinyCircos‐V1 has received frequent user feedback regarding bug reports and feature requests, most of which have been resolved. In this paper, we present shinyCircos‐V2.0, a significantly upgraded version of shinyCircos. The graphical user interface has been completely redesigned to provide a better user experience. Moreover, shinyCircos‐V2.0 has broken the limitations of a maximum of 12 input data sets, which was a restriction in shinyCircos‐V1. Additionally, advanced features such as color legends, text labels for track index, axis labels, and ticks have been implemented in shinyCircos‐V2.0. In comparison to its predecessor [10], shinyCircos‐V2.0 has been developed with improved usability and advanced features.

## RESULTS

### Refined graphical interface of shinyCircos‐V2.0 for enhanced user experience

**Figure 2:**
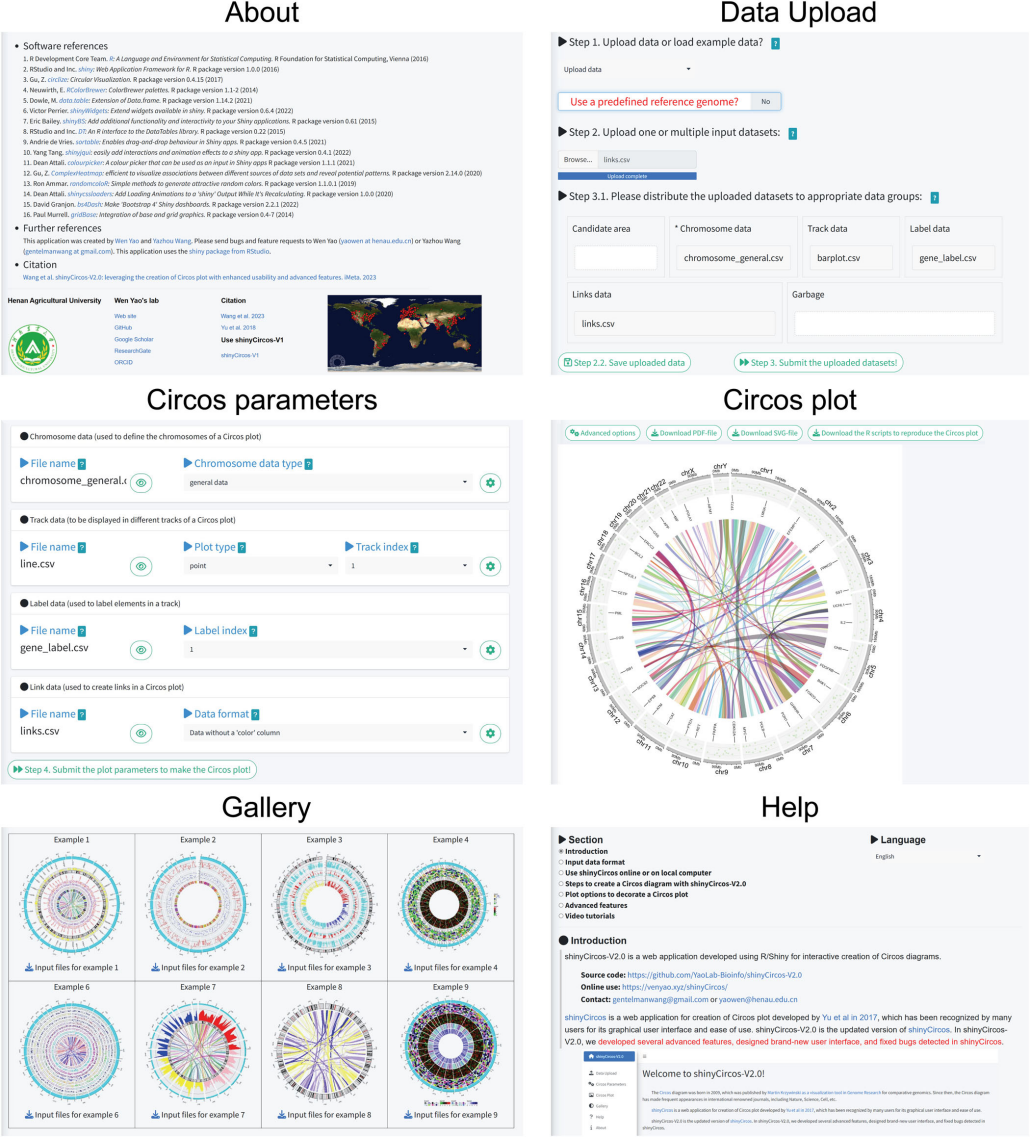
Main functionalities implemented on different pages of the shinyCircos‐V2.0 application. The title of each page is shown at the top.

In shinyCircos‐V2.0, we used the R package bs4Dash to develop an enhanced graphical user interface, which significantly improved the user experience [13]. bs4Dash is an updated version of the shinydashboard R package and provides dashboard templates for developing modern dashboards using Bootstrap 4 for R/Shiny applications. To facilitate the creation of Circos plots, we designed multiple pages, namely, the “Data Upload,” “Circos Parameters,” and “Circos Plot” pages, to organize the functionalities into different steps in sequential order (Figure 2). On the “Data Upload” page, users can upload one or more input data sets or choose to load an example input data set. Next, the input data sets are displayed in different sections on the “Circos Parameters” page. After setting the appropriate parameters for each input data set, the user can submit all input data sets and their parameters to create a Circos plot, which is then displayed on the “Circos Plot” page (Figure 2).

We have designed several additional pages to supplement the major functionalities of shinyCircos‐V2.0. These pages provide helpful tutorials on how to use the software effectively. The “Gallery” page showcases 30 example Circos plots created using shinyCircos‐V2.0, along with their corresponding input data sets (Figure 2). This serves to help users understand the proper format for input data sets when using shinyCircos‐V2.0. A detailed tutorial is available on the “Help” page in both English and Simplified Chinese. Additionally, the “About” page lists the dependent R packages used by shinyCircos‐V2.0. For bug reports and feature requests, users can find the relevant contact information on the “Contact” page.

In shinyCircos‐V1, all the widgets for data uploading were packed into the sidebar panel of the “Data Upload” page. While only the first few lines of each input data set were displayed in the main panel of the “Data Upload” page, resulting in an excessively long “Data Upload” page when multiple input data sets were uploaded. Similarly, all the parameters designed for each input data set were packed into the sidebar panel of the “Circos Visualization” page in ShinyCircos‐V1, making this page cumbersome if multiple input data sets were uploaded. Furthermore, each input data set had to be uploaded separately in ShinyCircos‐V1. By contrast, ShinyCircos‐V2.0 allows for the simultaneous upload of multiple input data sets, and the complete content of each input data set can be viewed in a pop‐up window on the “Circos Parameters” page (Figure 2). The plot parameters for each input data set were placed in separate pop‐up windows on the “Circos Parameters” page.

In ShinyCircos‐V2.0, we have included several additional pop‐up windows to enhance user interaction. Most of these pop‐up windows are triggered when incorrect input data sets or parameters are submitted. These windows provide prompt messages to assist users in utilizing ShinyCircos‐V2.0. Additionally, we have incorporated pop‐up windows with guiding information to aid users in the creation of Circos plots using ShinyCircos‐V2.0. Overall, ShinyCircos‐V2.0 features an improved user interface, making it much tidier and more user‐friendly than ShinyCircos‐V1.

### Seamless functionality integration in sequential pages of shinyCircos‐V2.0 for effortless Circos plot creation

We have deployed shinyCircos‐V2.0 online for users to access at https://venyao.xyz/shinyCircos/ and https://asiawang.shinyapps.io/shinyCircos/. For users who prefer offline usage, shinyCircos‐V2.0 can also be installed on their local computers. Please note that shinyCircos‐V1 is still hosted on our server at https://venyao.xyz/shinyCircos-V1/.

**Figure 3:**
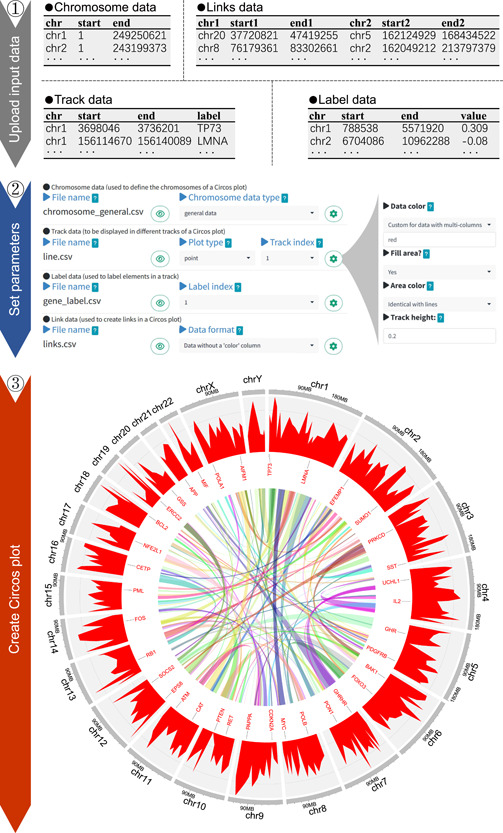
Illustration of the basic steps for creating a Circos plot with shinyCircos‐V2.0. The top panel displays the first two rows and column titles of the four input data sets. The middle panel showcases the widgets used to set parameters for each input data set. For each input data set, additional widgets can be accessed via a pop‐up window that can be opened by clicking on the gear symbol in the same line as the input data set. The bottom panel demonstrates the Circos plot created using the four input data sets and the set parameters.

The first step in utilizing shinyCircos‐V2.0 is to upload one or multiple input data sets using the widgets provided on the “Data Upload” page (Figure 3). Unlike shinyCircos‐V1, which had a maximum limit of 12 input data sets, shinyCircos‐V2.0 allows any number of input data sets to be uploaded, in principle. All the input data sets required to create a Circos plot can be uploaded simultaneously or separately. An indispensable input data set for a Circos plot is a Chromosome data set, which is used to define the length of each chromosome/scaffold in a genome. In shinyCircos‐V2.0, 20 Chromosome data sets are provided for model organisms, including humans, mice, *Arabidopsis*, Rice, and so forth. Other input data sets for a Circos plot can be grouped as Track data, which will be plotted alongside all the chromosomes/scaffolds, Label data to mark elements in different tracks with text labels, and Links data to make links between pairs of genomic regions. Before submitting the input data sets to the server‐side of shinyCircos‐V2.0, users should distribute all the input data sets into their appropriate data groups using the widgets provided on the “Data Upload” page. Furthermore, users can update the input data sets for a Circos plot by adding new data sets or deleting uploaded data sets, which can be useful for updating a created Circos plot without the need to reupload all the input data sets. It is important to save and submit all the input data sets once one or more of them have been updated.

After submitting all input data sets, the user is directed to the “Circos Parameters” page, which is divided into four areas to display the Chromosome data, Track data, Label data, and Links data (Figure 3). Each input data set is displayed on a single line, showing the filename, widgets to view the content, and other widgets to set plot parameters. For each track data, an appropriate plot type must be selected from 10 options, including points, lines, bars, rectangles with discrete values, rectangles with gradual values, heatmaps with discrete values, heatmaps with gradual values, chromosome ideograms, stacked points, and stacked lines. Most other parameters for all types of input data sets work smoothly with the default values assigned by shinyCircos‐V2.0. Nevertheless, the “Circos Parameters” page also includes many plot parameters that can be adjusted to decorate the Circos plot, such as the height of each track, the distance between adjacent tracks/sectors, the color of different bars/lines, the background color of each track, and the font size of text labels. For scatter points, the color, size, and shape of all points can be adjusted using the parameters implemented in shinyCircos‐V2.0. All input data sets and corresponding plot parameters must be submitted on the “Circos Parameters” page to create the desired Circos plot.

The Circos plot generated by shinyCircos‐V2.0 is finally displayed on the “Circos Plot” page (Figure 3). Users can download the Circos plot in high‐quality PDF or SVG format, which can be easily converted to other image formats, such as JPEG, PNG, TIFF, and so forth. Additionally, there are four advanced plot parameters available under the “Advanced Options” widget on the “Circos Plot” page. These parameters enable users to display the figure legend, track index labels, zoom in on the Circos plot, and highlight specific genomic regions within the plot. To improve the scalability of shinyCircos‐V2.0, we further provide the R scripts on the “Circos Plot” page for users to download and reproduce the Circos plot.

### New features implemented in shinyCircos‐V2.0 for the creation of advanced Circos plot

**Figure 4:**
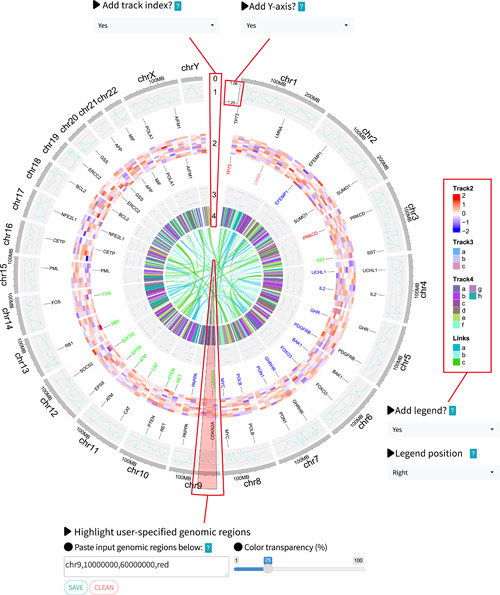
Demonstration of advanced features implemented in shinyCircos‐V2.0. Four new advanced features of shinyCircos‐V2.0 are highlighted with red boxes. Corresponding widgets to adjust parameters for these advanced features are positioned around the Circos plot.

To further enhance the capabilities of shinyCircos, we have implemented several advanced features in shinyCircos‐V2.0. For instance, shinyCircos‐V2.0 now supports proportionate zooming of the created Circos plot. Text labels added by Label data can be easily customized by tuning the font size and color, which can help distinguish different groups of elements in the corresponding track. Furthermore, shinyCircos‐V2.0 allows the addition of axis labels and ticks indicating the maximum and minimum values of the input data to bars, points, and lines created with track data (Figure 4). This feature is particularly useful for comparing input data sets displayed in different tracks. The index of different tracks is also important for interpreting the Circos plot, which was usually manually added to a Circos plot created using traditional Circos tools. In shinyCircos‐V2.0, users can now easily add text labels to indicate the index of all tracks (Figure 4).

Heatmaps are a ubiquitous type of plot in Circos diagrams used to depict expression patterns of multiple genes across different developmental stages/tissues. However, in shinyCircos‐V1, heatmaps can only be generated with numeric input data. In contrast, shinyCircos‐V2.0 supports the creation of heatmaps with both numeric and categorical input data. Furthermore, while color legends are essential for heatmaps, most existing Circos visualization software provides inadequate support. To overcome this limitation, we utilized the ComplexHeatmap [14] package to incorporate a color legend in shinyCircos‐V2.0. The color legend can be conveniently placed at the bottom or right side of the Circos plot for various plot types, such as “rect‐discrete,” “rect‐gradual,” “heatmap‐discrete,” “heatmap‐gradual,” “stack‐point,” “stack‐line,” and links (Figure 4).

Links are frequently used in Circos plots to represent interactions between pairs of genomic regions. For instance, genomic translocations can be depicted by links in a Circos plot. However, when connecting small genomic regions in a very large genome, links are often difficult to discern. This can be frustrating for users working on very large genomes, such as the wheat genome [15]. To address this issue, the circlize R package draws links using the middle points of each region, regardless of the size of the regions [4]. This feature has been incorporated into shinyCircos‐V2.0. Additionally, we have added new functionality to assign colors to different groups of links.

### Tutorials and exemplary input data sets for facilitating usage of shinyCircos‐V2.0

Being an intuitive and interactive web application, shinyCircos‐V2.0 is designed to be user‐friendly. To further enhance the user experience, we have included a comprehensive set of tutorials and example data sets, enabling users to effortlessly generate and customize Circos plots to meet their research needs.

Ten sets of examples of input data are provided on the “Data Upload” page of shinyCircos‐V2.0, which can be loaded and submitted to the platform with a single mouse click. For each example data set, the data group of each input data, the track index, the plot type of each input data, and the parameters for each input data, were preconfigured. Users can view these preconfigured parameters on the “Circos Parameters” page. Submitting the example data set and parameters generates a Circos plot on the “Circos Plot” page. These 10 sets of examples of input data produce the first 10 Circos plots displayed on the “Gallery” page of shinyCircos‐V2.0. The input data for each Circos plot can be viewed and downloaded on the “Data Upload” page, which can also be downloaded from the “Gallery” page. This feature is particularly convenient for new users to get started with shinyCircos‐V2.0.

On the “Gallery” page, a total of 30 Circos diagrams are displayed. The PDF file for each Circos plot can be accessed by clicking on the corresponding image on the “Gallery” page. The input data sets for each Circos plot can be downloaded using the download button below each Circos plot. The 30 Circos plots cover all aspects and features of the Circos plot created using shinyCircos‐V2.0, which is very helpful for users to prepare the input data sets and explore the functionalities of shinyCircos‐V2.0.

On the “Help” page of shinyCircos‐V2.0, we have provided a detailed tutorial in both Simplified Chinese and English. Additionally, we offer a table of contents to help users navigate different sections of the tutorial with ease. The tutorial comprises an introduction to shinyCircos‐V2.0 and the components of a typical Circos plot, the detailed format of each input data set required for shinyCircos‐V2.0, the step‐by‐step process to create a Circos plot using shinyCircos‐V2.0, as well as an explanation of plot parameters and advanced features implemented in shinyCircos‐V2.0. Furthermore, the help tutorial has been deposited in GitBook (https://yaolab-bioinfo.gitbook.io/shinycircos/) for browsing and sharing.

We have additionally produced 10 videos showcasing the process of creating the first 10 Circos plots that are displayed on the “Gallery” page of shinyCircos‐V2.0. These 10 videos have been uploaded to YouTube, NetEase Videos, and our own server, and the links to all of them have been provided on the home page of shinyCircos‐V2.0 on GitHub (https://github.com/YaoLab-Bioinfo/shinyCircos-V2.0) and the “Help” page of shinyCircos‐V2.0.

## CONCLUSION

With the advancement of sequencing technology, Circos graphs have become an essential tool for data visualization in various academic studies. Therefore, an easy‐to‐use tool with rich features is imperative. ShinyCircos‐V2.0 represents a significant improvement over the previous version of our application, featuring a more user‐friendly interface and many new features that enable users to create more advanced and customized Circos plots. Compared to ShinyCircos‐V1, ShinyCircos‐V2.0 offers additional features, such as track indexing, figure legends, axis labels, and ticks, as well as the capability to create an arbitrary number of tracks for Circos plots. We are committed to maintaining and improving shinyCircos‐V2.0, by resolving user feedback and implementing new enhancements. We believe that shinyCircos‐V2.0 will prove to be a valuable tool for researchers across diverse fields, ranging from genomics to ecology and beyond.

## AUTHOR CONTRIBUTIONS

Wen Yao conceived the study. Wen Yao, Yazhou Wang, and Lihua Jia designed the functions and the interfaces of the application. Yazhou Wang wrote the R code, wrote the help tutorials, and evaluated the application with help from Wen Yao, Lihua Jia, Ge Tian, Yihan Dong, Xiao Zhang, Zhengfu Zhou, Xiang Luo, and Yang Li. Wen Yao, Yazhou Wang, and Lihua Jia wrote the manuscript. All authors have read the final manuscript and approved it for publication.

## CONFLICT OF INTEREST STATEMENT

The authors declare no conflict of interest.

## Data Availability

The source code of shinyCircos‐V2.0 is available in GitHub (https://github.com/YaoLab-Bioinfo/shinyCircos-V2.0). Supporting Information (figures, tables, scripts, graphical abstract, slides, videos, Chinese translated version, and update materials) may be found in the online DOI or iMeta Science http://www.imeta.science/.
